# Potential Nutritional and Metabolomic Advantages of High Fat Oral Supplementation in Pancreatectomized Pancreaticobiliary Cancer Patients

**DOI:** 10.3390/nu11040893

**Published:** 2019-04-20

**Authors:** Bo Kyeong Yun, Mina Song, Ho Kyoung Hwang, Hosun Lee, Song Mi Lee, Chang Moo Kang, Seung-Min Lee

**Affiliations:** 1 Department of Food and Nutrition, Brain Korea 21 Plus Project, College of Human Ecology, Yonsei University, Seoul 03722, Korea; bogoming@naver.com (B.K.Y.); mina.song950@gmail.com (M.S.); 2 Division of Hepatobiliary and Pancreatic Surgery, Department of Surgery, Yonsei University College of Medicine, Yonsei Pancreatobiliary Cancer Center, Severance Hospital, Seoul 03722, Korea; DRHHK@YUHS.AC; 3 Department of Nutrition Care, Severance Hospital, Yonsei University Health System, Seoul 03722, Korea; HSLEE0730@yuhs.ac (H.L.); NUTRPINE@yuhs.ac (S.M.L.)

**Keywords:** postoperative oral nutritional supplement, high fat supplement, pancreatic cancer, metabolomics

## Abstract

We examined the effect of high fat oral nutritional supplement (HFS) on the nutritional status, oral intake, and serum metabolites of postoperative pancreaticobiliary cancer patients. Pancreaticobiliary cancer patients were voluntarily recruited. The HFS group received postoperative oral high fat supplementation (80% of total calories from fat; *n* = 12) until discharge; the control group (non-HFS; *n* = 9) received none. Dietary intake, anthropometry, blood chemistry, nutritional risk index (NRI), and serum metabolites analyzed by liquid chromatography tandem mass spectrometry were evaluated. Overall, cumulative caloric supply via parental and oral/enteral routes were not different between groups. However, oral fat intake, caloric intake, and NRI scores of the HFS group were higher than those of the non-HFS group with increased oral meal consumption. Oral caloric, fat, and meal intakes correlated with NRI scores. Metabolomics analysis identified 195 serum metabolites pre-discharge. Oral fat intake was correlated with 42 metabolites relevant to the glycerophospholipid pathway. Oral high fat-specific upregulation of sphingomyelin (d18:1/24:1), a previously reported pancreatic cancer-downregulated metabolite, and lysophosphatidylcholine (16:0) were associated with NRI scores. Provision of HFS in postoperative pancreatic cancer patients may facilitate the recovery of postoperative health status by increasing oral meal intake, improving nutritional status, and modulating serum metabolites

## 1. Introduction

Postsurgical malnutrition is frequently observed in pancreatic cancer patients [1]. Pancreatic resection-associated complications, such as pancreatic fistula, fluid collection within the abdomen, and delayed gastric emptying, can reduce oral intake and increase the risk of malnutrition, subsequent weight loss, and delayed hospital discharge [2,3,4]. Postoperative nutritional support reduces surgical complications especially in malnourished gastrointestinal (colorectal, stomach, or pancreatic) cancer patients [5]. Nutritional support using parenteral nutrition, enteral nutrition, and an oral diet reduced the levels of postoperative inflammatory indicators, including C-reactive protein (CRP) [6]. The Enhanced Recovery After Surgery Society recommended that the provision of an oral diet immediately after pancreaticoduodenectomy is a feasible and desirable form of nutritional support for pancreatic cancer patients [7]. When oral intake cannot meet the necessary nutritional requirement, oral nutrition supplement (ONS) can be used for stabilizing the body weight and body composition [8]. An ad libitum ONS has been demonstrated to improve dietary intake, reduce weight loss, and decrease the complication rate in patients who underwent gastrointestinal surgery [9]. An oral feeding protocol utilizing ONS reduced the length of stay by 6 days in postoperative patients compared with routine enteral tube feeding [10]. A significant reduction in body weight was also reported in severely malnourished patients who received the ONS [11]. Thus, the use of ONS to aid an oral diet may improve the recovery of pancreatic cancer patients after surgery.

Commercially available ONS has 20% to 30% of its total calories from fat [12] and energy densities ranging from 1 to 2 kcal/mL [13]. Fat primarily serves as an energy source for cells apart from cellular components and signaling molecules [14]. Fat supply in postoperative patients increases energy supply and provides essential fatty acids to prevent essential fatty acid deficiency and high glucose infusion rate, thus decreasing the risk of hyperglycemia [15]. Keim et al. recommended an increase in dietary fat content with administration of pancreatic enzymes to increase caloric density in the diet and provide lipid soluble vitamins for postoperative pancreatic cancer patients [16]. Increasing fat content in ONS may increase caloric density in malnourished patients, including postoperative patients [17]. The administration of supplements with an energy density higher than 1 kcal/mL could lower total intake volume and lead to good compliance, especially in malnourished patients [13]. In addition, dietary fat may stimulate oral intake due to high palatability and weak satiety effect [18]. 

Here, we hypothesized that the use of an easily digestible medium chain triglyceride (MCT)-containing high fat oral nutrition supplement could improve the nutritional status in postoperative pancreatic cancer patients. We aimed to investigate the effects of oral nutrition supplement on the nutritional status using the nutritional risk index (NRI), oral food intake, blood biochemical parameters, and serum metabolites in pre- and postoperative pancreatic cancer patients in comparison to a general hospital diet. 

## 2. Materials and Methods

### 2.1. Study Participants

A total of 25 pancreaticobiliary cancer patients (aged ≥19 years) at the Severance Hospital pancreatic surgery clinic (Seoul, Korea) from September 2017 to January 2018 were recruited for this study. All subjects agreed and were informed before participating in the study. Written informed consent was obtained prior to their participation. Patients with pancreatic, duodenal, distal bile duct, and ampullary cancers were included. Pregnant women; individuals who developed severe diabetes with complications, hyperlipidemia with cardiovascular complications, or renal insufficiency; and illiterate patients were excluded. After screening for eligibility and excluding dropouts, 12 patients were assigned to the experimental group (HFS, high fat supplementary formula) and 9 to the control group (non-HFS, no supplementary formula) for the study analysis. This study proceeded according to the Declaration of Helsinki and was approved by the Severance Hospital institutional review board (approval number: 4-2017-0625) (Seoul, Korea), and registered in ClinicalTrials.gov (NCT03294096).

### 2.2. Dietary Intervention

**Figure 1:**
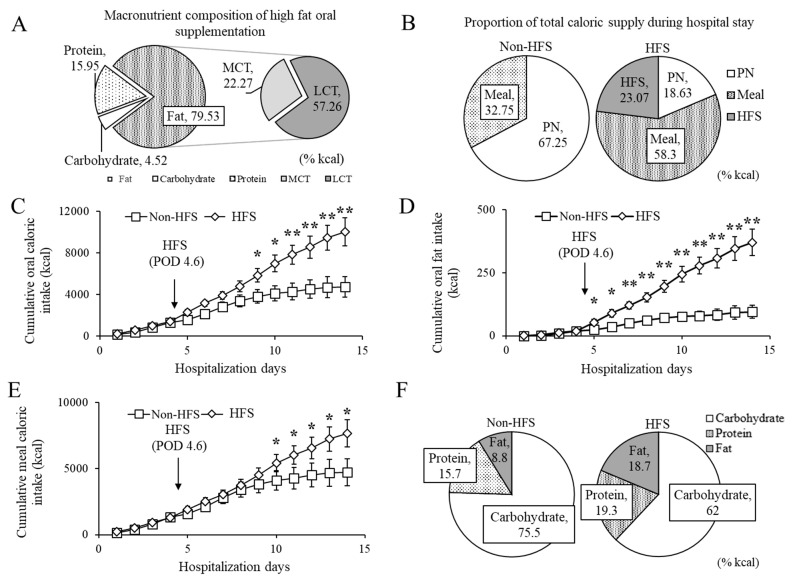
Nutritional composition of high fat oral supplement (HFS) and cumulative intake calories in postoperative pancreatic cancer patients. (**A**) Macronutrient composition of high fat oral supplementation (%kcal); (**B**) proportion of total caloric supply during hospital stay; (**C–F**) cumulative oral caloric intake, cumulative oral fat intake (kcal), cumulative meal caloric intake, and proportion of oral macronutrient composition by postoperative days during the entire hospitalization period. Total supply included parenteral nutrition and meal (non-HFS) or parenteral nutrition, meal, and oral supplement (HFS). Oral intake included meal intake (non-HFS) or meal and supplement intake (HFS). Meal intake considered only meal intake, excluding formula intake. All values were expressed as mean ± standard error. Statistical differences determined by independent student *t*-test * *p* < 0.01; ** *p* < 0.05. MCT, medium-chain triglycerides; LCT, long-chain triglycerides; non-HFS, non-supplemented group; HFS, high fat supplement group; PN, parenteral nutrition; POD, postoperative day.

During the postoperative period, all patients received standard care. The non-HFS and HFS groups received a 100% carbohydrate oral supplement (Daesang Wellife, Seoul, Korea) from postoperative day (POD) 1, which was replaced by full liquid diet after 3 to 4 days and soon after by a soft diet. Then non-HFS patients received a 1500 kcal general hospital diet without any supplement. On the other hand, when the HFS group was allowed a full liquid diet, three packs of an oral supplement (HFS) were provided each day until discharge. The HFS was 125 mL in volume, providing 150 kcal in energy (Carbohydrate: Protein: Fat = 4: 16: 80, % kcal). Fats comprised 22.3% of MCT and 57.3% of high oleic acid sunflower oils in caloric content. The detailed composition of the macronutrients and fatty acids are listed in Figure 1A and Supplementary Table S1. Parenteral nutrition of a lipid emulsion, 500 mL of Smoflipid 20% (Fresenius Kabi, Linz, Austria), and if necessary, additional administration of 10% dextrose water and/or Winuf^®^ peri (JW, Seoul, Korea) 1450 mL (113 g glucose, 46 g protein, and 41 g fat per 1450 mL) was administered only to the non-HFS group 1 to 4 days before discharge.

### 2.3. Dietary Assessment

On the day before surgery (baseline), the patient’s regular dietary intake was assessed by dietitians via 24-h recall. During the hospital stay, daily dietary intake was self-recorded. Dietitians conducted one-on-one interviews with the patients or their guardians on daily food intake until the day before discharge (preDC). After discharge (postDC), food intake records written by patients for 5 to 15 days were used for dietary intake assessment. Energy, carbohydrate, protein, and lipid intakes were assessed using the computer software, Can-Pro 4.0 (Computer Aided Nutritional Analysis Program for Professionals, the Korean Nutrition Society, Seoul, Korea). 

### 2.4. Anthropometry, Blood Chemistry, and Nutritional Status Measurement

Data regarding body weight, height, serum carcinoembryonic antigen (CEA), Carbohydrate antigen 19-9 (CA 19-9), prealbumin, albumin, creatinine, triglyceride, total cholesterol (Total-C), high-density lipoprotein cholesterol (HDL-C), low-density lipoprotein cholesterol (LDL-C), lipoprotein, transferrin, and CRP levels were collected from medical records. Ideal body weight was calculated using the Lorentz formula [19]. Estimated energy requirements were set for daily energy requirements based on the defined activity level [20]. NRI was calculated as follows: NRI = [15.19 × serum albumin (g/L)] + [41.7 × present weight (kg)/ideal body weight (kg)] [21]. Postoperative complications were assessed based on the criteria previously described by Buzby et al. [22]. 

### 2.5. Blood Collection and Metabolite Extraction 

Blood samples were collected after an overnight fast. The serum was separated and mixed with 8 times the volume of 70% methanol in acetone. Internal standard, a mixture of acetaminophen, sulfadimethoxine, terfenadine, and reserpine (Sigma-Aldrich, Oakville, ON, Canada), was then added to the serum mixture. After centrifugation at 10,000 rpm × 5 min at 4 °C, the supernatant was lysophilized for about 18 h. The lysophilized sample was resuspended in 10% methanol. After quick centrifugation, the resultant supernatant was collected in a glass vial and used for non-targeted metabolomic analysis. In order to maintain sample quality, quality control samples were prepared by pooling serum samples from all participants.

### 2.6. Liquid Chromatography Orbitrap Tandem Mass Spectrometry Analysis

Ultimate 3000 UHPLC and Q-Exactive Orbitrap Plus (Thermo Fisher Scientific, Waltham, MA, USA) equipped with a Fourier transform mass spectrometry (FTMS) analyzer was used for micro-liquid chromatography (LC). Waters columns (2.1 × 150 mm) packed with C18 stationary 1.7 μm-sized resins were used for chromatographic separations. MS data were acquired in full scan (80–1000 *m*/*z*) using data-dependent MS^2^ (dd-MS^2^) top 10 analysis in ESI-positive mode with a resolution of 70,000. To ensure data quality and for a reliability check, quality control samples were injected into every 10th sample. 

### 2.7. Data Processing and Analysis

The obtained LC-(ESI+)-MS/MS data in raw file format were initially processed with XCalibur 2.2 (Thermo Fisher Scientific, San Jose, CA, USA). Then, automatic detection and integration of peaks was performed via the pairwise job on the XCMS online software (http://xcmsonline.scripps.edu/) using the following parameters: Bandwidth (10 MHz), unpaired nonparametric method, database search tolerance (5 ppm), and UPLC/Orbitrap default settings. The resulting data included retention time, *m*/*z* ratio, and ion intensity. Data from XCMS were used to extract MS/MS peak intensities on XCalibur 2.2 according to query m/z and retention time. For metabolic identification, the online databases, HMDB (Human Metabolome Database, www.hmdb.ca/) and MycompoundID (www.mycompoundid.org), were used.

### 2.8. Statistical Analysis

Statistical analysis of general characteristic parameters between the groups was performed using an independent *t*-test and Mann-Whitney *U* test (SPSS version 23.0 software, Chicago, IL, USA). A paired *t*-test was used for within-group comparison analysis. The results were expressed as mean ± standard error (SE) with *p* < 0.05 representing statistical significance. For all metabolites, univariate Mann-Whitney *U*-test and multivariate orthogonal partial least squares discriminant analysis (OPLS-DA) were performed (using SMICA version 14.1, Umetrics Inc., Umea, Sweden). Logarithmic transformation and Pareto scaling were carried out before multivariate analysis. The goodness of fit was indicated by R^2^X and R^2^Y, and the predictive ability was assessed by Q^2^Y parameters. A 7 cross validation-analysis of variance and a permutation test (*n* = 500) were performed to validate the models. The meaningful metabolites were listed according to the following parameters, including a univariate *p*-value of <0.05 and multivariate variable importance in the projection (VIP) value of >1.0. Pearson correlation coefficient analysis (Pearson’s *r*) was performed to investigate whether metabolites correlated with the NRI score, caloric intake, and oral fat intake. 

## 3. Results

### 3.1. Baseline Clinical Characteristics and Oral Supplementation

No significant differences were observed in variables, including age, gender distribution, BMI, diagnosis, and type of operation between groups (Table 1). The average duration for oral diet was similar in both groups (Table 1). On average, full liquid diet in the non-HFS and HFS groups was started on POD 1.7 ± 0.7 and 1.3 ± 0.2, respectively. Full liquid diet was started on POD 4.3 ± 0.8 in the HFS group along with oral high fat supplement and on POD 4.6 ± 0.4 in the non-HFS group without the supplement. On average, 33.3% of HFS patients had more than 2 cans per day of HFS protocol, and the remaining 66.7% of patients took HFS 1 to 2 times a day. A total of 64 ± 21.4 servings of HFS were consumed by patients throughout the study period. The HFS group experienced general side effects, including postoperative pancreatic fistula (POPF) (2), bowel ileus (1), anxiety and pain (1), bilirubin elevation (1), pancreatitis (1), and generalized edema (1). The non-HFS group had POPF, bile leak (1), wound infection (2), hematochezia (1), and pneumonia (1). Our high fat ONS formula did not exert serious postoperative complications. The average duration of hospital stay was not different between the groups (Table 1). 

### 3.2. Analysis of Oral Caloric Intake and Meal Intake

Total average daily caloric intake did not differ between the groups (1367.2 ± 75.5 kcal/day in the HFS group and 1255.4 ± 123.0 kcal/day in the non-HFS group, *p* = 0.485). The average oral caloric intake in the HFS group was higher than that in the non-HFS group (949.6 ± 88.7 kcal/day in the HFS and 447.8 ± 71.4 kcal/day in the non-HFS, *p* < 0.001). Approximately, the estimated energy requirement was achieved more by oral caloric intake in the HFS group than in the non-HFS group (Table 1). The non-HFS group had higher non-oral caloric intake (via parenteral nutrition) than the HFS group (*p* < 0.05, Figure 1b). From POD 9 to 14, the HFS group showed a higher cumulative oral caloric intake than the non-HFS group (Figure 1c). The HFS group had higher cumulative oral fat intake (kcal) on PODs 5 to 14 (Figure 1d). Interestingly, a high meal intake of 284.17 kcal was seen in the HFS group compared to the non-HFS group (not shown, *p* = 0.012). Cumulative meal caloric intake was higher on PODs 10 to 14 in the HFS group than in the non-HFS group (Figure 1e). When nutrition supply was considered only via the oral route, the HFS group was provided more fat and protein and less carbohydrates than the non-HFS group (Figure 1f). 

### 3.3. Anthropometry, Blood Biochemistry, NRI Score, and Pancreaticobiliary Cancer Blood Biomarkers

**Figure 2:**
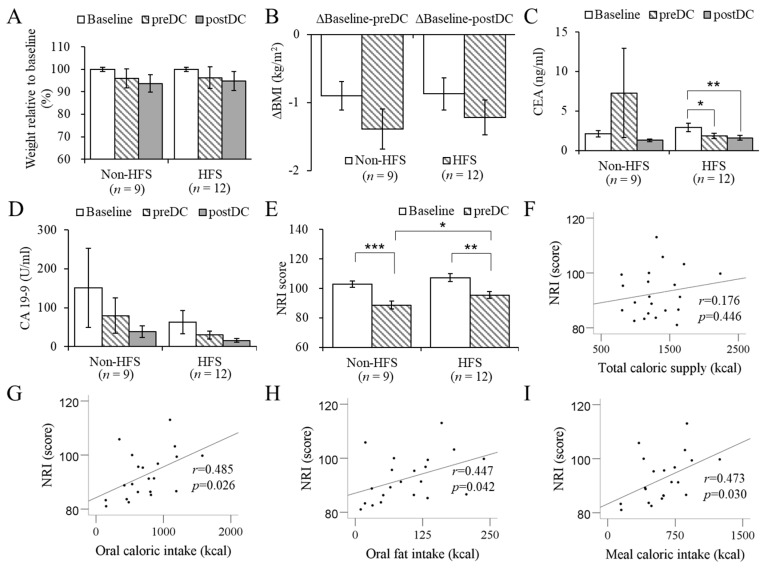
Changes in body weight, pancreatic blood biomarkers, and nutritional risk index (NRI) and the correlations between NRI scores and oral intakes of postoperative pancreatic cancer patients. (**A–B**) Body weight (ANOVA) and weight percentage relative to baseline (paired *t*-test) compared between groups at different time points; (**C–D**) blood levels of pancreatic cancer-specific biomarkers, carcinoembryonic antigen (CEA) and carbohydrate antigen (CA 19-9), at different time points in the non-HFS and HFS groups (Student’s *t*-test). (**E**) Changes in nutritional risk index (NRI) score on the day before discharge (preDC) compared between non-HFS and HFS groups (Mann-Whitney *U* test). (**F–I**) Correlation between NRI score and total caloric supply (**F**), oral caloric intake (**G**), oral fat intake in calories (**H**), and meal caloric intake (**G**) (Pearson’s *r* correlation analysis). Values expressed as mean ± standard error. Non-HFS, non-supplemented group; HFS, high fat supplement group; preDC, day before discharge; postDC, first outpatient visit day after discharge; BMI, body mass index; NRI, nutritional risk index; CEA, carcinoembryonic antigen; CA 19-9, cancer antigen 19-9. *p < 0.05, **p < 0.01, ***p < 0.005.

Postoperatively, both groups lost body weight at preDC and postDC than at the baseline, and the extent of weight loss was not significantly different between groups (Figure 2a–b). The changes in all tested blood biochemical data, including serum albumin, Total-C, HDL-C, and LDL-C, were not significantly different between the non-HFS and HFS groups at preDC and postDC compared to the baseline (Table 2.) NRI is a valid malnutrition evaluation tool and has been proven effective in the assessment of malnourished patients who underwent pancreatic surgery [23]. NRI scores in both groups significantly decreased at preDC compared to the baseline, but the score was significantly higher in the HFS groups than in the non-HFS group (Figure 2e). NRI scores were correlated with oral caloric intake (kcal), oral fat intake (g), and meal caloric intake (kcal) (Figure 2g–I, *p* < 0.05), but not with total caloric supply (Figure 2f).

### 3.4. Analytical Model Selection for Non-Targeted Metabolomics

**Figure 3:**
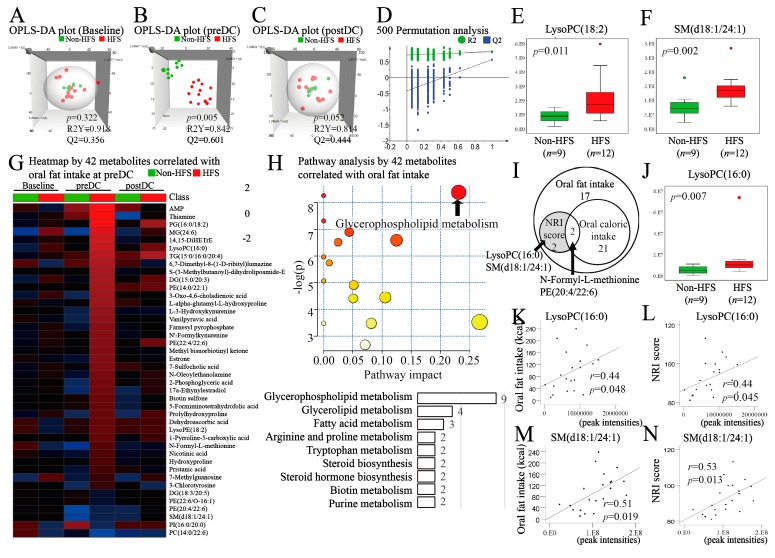
Metabolic analyses of oral fat intake-associated serum metabolites and their correlation with NRI score. (**A–C**) Orthogonal partial least squares discriminant analysis (OPLS-DA) score plot comparing HFS and non-HFS groups at baseline (**A**), preDC (**B**), and postDC (**C**); (**D**) 500 permutation validation plot for non-HFS compared with HFS at preDC. All permuted *R*^2^ and *Q*^2^ values on the left were lower than the points on the right, and *Q^2^* regression line had a negative intercept (*R*^2^ = 0.776, *Q*^2^ = −0.428, *Y* = −0.542); (**E–F**) box plots for dysregulated pancreatic cancer-specific metabolites, log-transformed; (**G**) heat map for comparison by 42 metabolites correlated with oral fat intake in the HFS group compared with the non-HFS group at baseline, preDC, and postDC. The values used in the heat map are log_2_ transforms of fold change values divided by baseline peak intensities; (**H**) pathway diagram of 42 oral fat correlated-metabolites significantly hit in the pathway analysis. (**I**) Venn diagrams reflecting metabolites significantly correlated in oral fat intake (*n* = 42), oral caloric intake (*n* = 23), and NRI scores (*n* = 4) among 195 metabolites; (**K–N**), LysoPC(16:0) and SM(d18:1/24:1) significantly correlated with oral fat intake and NRI score (Pearson’s *r* correlation analysis). OPLS-DA, orthogonal partial squares discriminant analysis; non-HFS, non-supplemented group; HFS, high fat supplement group; preDC, day before discharge; postDC, first outpatient visit day after discharge; LysoPC, lysophosphatidylcholine; SM, sphingomyelin; PE, phosphatidylethanolamine; NRI, nutritional risk index.

To identify the serum metabolites affected by HFS, non-targeted metabolomic analysis was performed. A total of 10,416 ionized compounds were detected from the MS/MS data of serum samples at the baseline, preDC, and postDC. Metabolomic models of group comparison between HFS and non-HFS samples were validated by OPLS-DA only at preDC (Figure 3a–c). Figure 3a and c showed no significant differences in the metabolites of the two groups obtained at the baseline and postDC (*p* = 0.322 and *p* = 0.052). The only adaptable model was the comparison between the two groups at preDC (R^2^Y = 0.842, Q^2^ = 0.601, *p* = 0.005, Figure 3b). A 500 permutation analysis validated the analytical model (Figure 3d). 

### 3.5. Identification of Metabolites Present in the HFS Group and the Non-HFS Group

After comparison of metabolites at preDC of the non-HFS and HFS groups, 516 differential peaks were selected using the cutoff values of the OPLS-DA VIP score of >1.0 and *p* value of <0.05. In all, 321 peaks were excluded due to non-identification from the libraries or being identified to be drugs and xenobiotics. Finally, 195 metabolites were identified and used for further analysis (Supplementary Table S2). When compared with the non-HFS group, 176 metabolites were up-regulated and 19 metabolites were down-regulated in the HFS group (Supplementary Figure S1). Among previously reported pancreatic cancer-associated metabolites [24], significant upregulations in lysophosphatidylcholine (LysoPC)(18:2) (Figure 3e) and sphingomyelin (SM)(d18:1/24:1) (Figure 3f) were detected in the HFS group compared with the non-HFS group. A significant upregulation of oleic acid in the HFS group was detected compared to the non-HFS group (Supplementary Table S2). 

### 3.6. Identification of Metabolites Associated with both NRI and Oral Fat Intake

Oral fat intake was correlated with 42 metabolites (Supplementary Table S3). Heat maps of these metabolites at the baseline, preDC, and postDC are shown in Figure 3g. The significant metabolic pathway associated with them was that of glycerophospholipid metabolism (false discovery rate (FDR) = 0.002, pathway impact = 0.231) (Figure 3h). Thirteen out of 42 metabolites were related to glycerophospholipid metabolism (9 metabolites) and to glycerolipid metabolism (4 metabolites). Because of the significant correlation between oral fat intake and the NRI score (Figure 2f), we further identified which oral fat intake-correlated metabolites were also associated with the NRI score. Of the 42 metabolites, 4 were associated with changes in NRI scores (Figure 3i), which were *N*-formyl-l-methionine, phosphatidylethanolamine (PE)(20:4/22:6), LysoPC(16:0), and SM(d18:1/24:1). Among these 4 metabolites, oral fat-specific correlation was seen only with LysoPC(16:0) and SM(d18:1/24:1) (Figure 3i,k–n). 

## 4. Discussion

This was the first study to report that high fat-derived energy dense ONS application was tolerable and feasible in pancreatic cancer patients who underwent pancreatobiliary resection and could further improve patients’ nutritional statuses. Throughout the study period, the subjects demonstrated fair levels of compliance to HFS in comparison with other ONS studies on postoperative patients [13]. We expected higher levels of total caloric intake in the HFS group than in the non-HFS group because of additional oral supplement. However, high energy supply via parental nutrition in the non-HFS group resulted in no significant differences in total calorie intake between groups. A conspicuous effect of the HFS was the enhancement of oral caloric intake via the increase in meal intake and HFS. Interestingly, oral fat or calorie intake was associated with NRI scores, suggesting that the oral high fat supplementation might have improved the nutritional status in postoperative pancreatic cancer patients.

Our fat source was mainly high oleic acid sunflower oil and MCT, which gave rise to 38.0% oleic acid and 22.3% MCTs of the total calories in the supplement. Anti-cancer effects of oleic acid have been reported in many types of cancers, including pancreatic cancer [25], breast cancer [26], and colorectal cancer [27]. In a prospective cohort study, dietary oleic acid intake was inversely related to the risk of pancreatic ductal adenocarcinoma (significant in patients with a BMI > 25 kg/m^2^) [25]. Another study using tumor tissues of pancreatic ductal adenocarcinoma patients revealed that oleic acid and stearic acid were significantly higher in the group with favorable survival than in that with low survival [28]. Thus, our HFS might have provided additional benefits to the patients due to the high contents of oleic acid. The use of MCT as a fat source in the HFS was intended to enhance intestinal absorption without further increasing the need for digestive enzymes. Because of their short fatty acid length, MCTs are absorbed via the portal vein and can be an efficient energy source compared with long-chain triglycerides [29,30]. The MCTs used in the study were caprylic (C8:0), capric (C10:0), and lauric (C12:0) acids. MCT may also spare muscle protein for energy demand in postoperative patients and possibly increase survival rate [31]. In a randomized clinical trial with gastrointestinal cancer patients who underwent surgery (including 30 patients who had pylorus-preserving pancreaticoduodenectomy), MCT supplementation was effective in increasing the plasma prealbumin and reducing the length of the hospital stay [32]. Although there were conflicting reports of MCT on appetite [33,34], the use of MCT in the HFS might have increased the meal consumption in our subjects who were in energy-demanding states due to postsurgical stress. MCT has been known to decrease dietary food [33]. However, a recent study reported the appetite-increasing effects of MCT in anorexia nervosa patients [34]. MCT (>6 g/day) increased the appetite and hormone ghrelin levels in these anorexic patients [34]. High fat itself was also suggested to increase caloric intake [35]. A high fat diet for 2 weeks increased the food intake and body weights of healthy individuals [36]. Exposure to a high fat meal increased energy intake after 24 hours compared with high carbohydrate meal [37]. Increased food intake after lipid ingestion has been demonstrated due to orosensory stimulation in rats [38]. Oral consumption of fat may slow satiety signals than direct fat delivery into the intestines, therefore allowing higher calorie intake before reaching satiety [18]. By contrast, fat effects on satiety can differ depending on the extent of saturation and/or lengths of fatty acids. Monounsaturated (oleic acid, C18:1) and saturated fats (stearic acid, C18:0) had less satiety effects than polyunsaturated fats (linolenic acid, C18:2) [37]. Thus, more research on the effects of HFS on appetite and/or meal intake in patients at nutritional risk is warranted. 

Our metabolomic analysis using UHPLC-Orbitrap-FTMS found 42 potential metabolite biomarkers associated with oral high fat supplementation in postoperative pancreatic cancer patients. Hit metabolites were involved in lipid metabolism (glycerophospholipid, glycerolipid, fatty acid metabolism, etc.). Alterations in phospholipid levels have been reported in pancreatic cancer patients, in whom LysoPC(18:2) was downregulated [24], while upregulation of this metabolite was detected in the HFS group compared to the non-HFS group. Another study demonstrated the inverse relationships between the levels of LysoPC (16:0, 18:2, and 20:4) metabolites and the risk of breast, prostate, and colorectal cancer [39]. All LysoPCs differentially detected in our HFS group were upregulated, which were LysoPC(16:0, 18:2, 18:3, 20:0, 20:1, 20:2, 20:4, 22:5, 22:6, and 24:1). Particularly, LysoPC(16:0) was positively associated with NRI scores. In a mass spectrometry-based study, plasma LysoPC(16:0) was more significantly decreased in lung cancer patients than in healthy controls [40]. In particular, low levels of LysoPC(16:0) in cancer tissues were suggested to be a biomarker associated with recurrence in prostate cancer patients [41]. Oral high fat supplementation-induced upregulation of LysoPC metabolites, including LysoPC(16:0), might have yielded beneficial health effects in postoperative pancreatic cancer patients. On the contrary, high LysoPC(16:0) levels were reported to be an ovarian cancer biomarker [42]. Thus, lysophospholipid metabolism in cancer might vary depending on the type and stage of cancer. Another metabolite associated with both oral fat intake and NRI score was SM(d18:0/24:1). Eicosanoids generated by the action of phospholipase A_2_ (PLA_2_) can increase the enzymatic hydrolysis of sphingomyelinase and produce SM, a major constituent of lipid rafts in the plasma membrane [43]. The same SM(d18:0/24:1) metabolite was downregulated in pancreatic cancer patients [24]. Exogenous sphingomyelin inhibits chemokine-induced cell migration [44]. The upregulation of SM(d18:0/24:1) by oral high fat supplementation might have contributed to the overall health benefits of the HFS in postoperative pancreatic cancer patients, but further studies are warranted to confirm this finding. 

We demonstrated that oral high fat supplementation in postoperative pancreatic cancer patients increased their oral caloric and meal intake and thereby improved NRI. Serum metabolites associated with pancreatic cancer were altered in benefit of the surgical patients receiving high fat oral supplementation compared with the control group. In conclusion, oral high fat supplementation may have positive effects on the health status of postoperative pancreaticobiliary cancer patients. 

## Figures and Tables

**Table 1:** Comparison of baseline clinical characteristics and energy intake during hospital stay between non-HFS and HFS groups.

Patient Characteristics	Non-HFS (*n* = 9)	HFS (*n* = 12)	*p*-Value
Age (year)	66.3 ± 3.3	55.2 ± 4.4	0.095
Male/Female (*n* (%))	6 (66.7)/3 (33.3)	7 (58.3)/5 (41.7)	0.714
Weight (kg)	56.3 ± 7.3	65.1 ± 10.7	0.037
PIBW (kg/kg%)	103.2 ± 3.9	112.4 ± 4.0	0.123
BMI (kg/m^2^)	22.2 ± 0.9	23.7 ± 0.8	0.236
Histological type			
Pancreatic ca. (head/body/tail)	3 (1/1/1)	5 (3/1/0)	
Ampulla of vater cancer	2	2	
Common bile duct	4	2	0.781
Pancreatic NET	0	1	
IPMN	0	1	
Solid pseudopapillary carcinoma	0	1	
Surgical operation			
PPPD	8	10	1.000
DP	1	2	
Total oral diet period (day)	14.2 ± 3.5	14.7 ± 2.8	0.922
Percentage of energy intake to EER (kcal/kcal %) *			
from total caloric intake (%)	89.6 ± 5.9	75.7 ± 7.5	0.185
from oral intake (%)	28.5 ± 4.1	57.0 ± 5.3	0.001
from meal (%)	28.5 ± 4.1	43.9 ± 4.2	0.003
Hospitalization period	14.9 ± 3.6	15.0 ± 2.7	0.980

*p*-values were derived from independent Student’s *t*-test at baseline. The statistical differences between groups for histological type were obtained using Fisher’s exact test after crossover analysis. * measured during hospitalization period. Values were expressed as mean ± standard error. PIBW, percentage weight to ideal body weight; BMI, body mass index; pancreatic NET, pancreatic neuroendocrine tumors; IPMN, intraductal papillary mucinous neoplasm; PPPD, pylorus-preserving pancreaticoduodenectomy; DP, distal pancreatectomy; EER, estimated energy requirement.

**Table 2:** Comparison of biochemical parameters between non-HFS and HFS groups.

Parameters	Normal Range	Non-HFS (*n* = 9)			HFS (*n* = 12)			*p*-Value		
		Baseline	preDC	postDC	Baseline	preDC	postDC	Baseline ^ǂ^	preDC ^§^	postDC ^§^
Prealbumin (g/L)	150–350	198.9 ± 20.4	153.3 ± 23.4	226.9 ± 23.0	238.7 ± 6.1	175.5 ± 14.8 *	242.7 ± 20.2	0.1450	0.9914	0.5753
Albumin (g/dl)	3.5–5.0	3.94 ± 0.13	3.12 ± 0.10 *	-	3.98 ± 0.16	3.32 ± 0.13 *	-	0.8842	0.3055	-
Creatinine (mg/dl)	10–300	65.4 ± 18.2	82.3 ± 15.2	150.2 ± 16.4 *	75.7 ± 10.7	91.7 ± 18.3	215.0 ± 34.9 *	0.6340	0.8618	0.1340
TG (mg/dl)	<150	120.8 ± 24.8	130.4 ± 22.4	110.6 ± 17.1	129.6 ± 11.9	125.5 ± 10.9	124.1 ± 6.8	0.7548	0.6614	0.4836
Total-C (mg/dl)	<200	155.8 ± 46.2	136.7 ± 30.4	156.0 ± 48.5	174.3 ± 45.2	137.8 ± 32.9 *	168.0 ± 42.6	0.1849	0.4683	0.2770
HDL-C (mg/dl)	≥60	43.9 ± 3.8	28.6 ± 3.4 *	46.9 ± 5.0	43.8 ± 3.2	33.5 ± 4.1 *	44.3 ± 3.8	0.9778	0.2872	0.6695
LDL-C (mg/dl)	<100	86.0 ± 12.2	81.3 ± 10.4	97.4 ± 14.8	107.2 ± 11.1	81.5 ± 8.1 *	107.1 ± 10.9	0.2146	0.0911	0.8857
Transferrin (mg/dl)	212–360	208.7 ± 17.8	181.4 ± 17.7 *	221.4 ± 14.2	252.8 ± 11.8	185.8 ± 21.5 *	255.5 ± 11.1	0.0580	0.3672	0.3146
Lipoprotein (mg/dl)	>30	19.98 ± 4.63	18.13 ± 4.69	26.18 ± 8.84	11.70 ± 2.5	15.69 ± 3.95	14.24 ± 2.90	0.1431	0.2699	0.8775
CRP (mg/L)	<3.00	14.13 ± 11.69	38.90 ± 8.72 *	10.04 ± 3.93	2.81 ± 0.78	32.68 ± 10.19 *	5.53 ± 2.66	0.3618	0.9883	0.7493

Values were expressed as mean ± standard error. ^ǂ^ Non-HFS and HFS groups were compared at the baseline using independent samples of Student’s *t*-test. ^§^The difference between the two groups at preDC and postDC was compared using linear regression with baseline as the control factor. *p*-values obtained for the statistical analysis in the group were derived from paired-independent Student’s *t*-test. * *p* < 0.05. TG, triglyceride; Total-C, total cholesterol; HDL-C, high-density lipoprotein cholesterol; LDL-C, low-density lipoprotein cholesterol; CRP, C-reactive protein.

## Supplementary Materials

The following are available online at https://www.mdpi.com/2072-6643/11/4/893/s1, **Figure S1.** Heatmap of total metabolites differentially identified in the comparison of non-HFS and HFS groups at preDC; **Table S1.** Fatty acid composition (%) of the high fat oral supplement; **Table S2.** List of total metabolites differentially identified in the comparison of non-HFS and HFS groups at preDC; **Table S3.** List of metabolites correlated with oral fat intake (kcal).

## References

[1] Hashimoto D., Chikamoto A., Ohmuraya M., Abe S., Nakagawa S., Beppu T., Takamori H., Hirota M., Baba H. (2015). Impact of postoperative weight loss on survival after resection for pancreatic cancer. J. Parenter. Enteral. Nutr..

[2] Di Carlo V., Gianotti L., Balzano G., Zerbi A., Braga M. (1999). Complications of pancreatic surgery and the role of perioperative nutrition. Digestive Surg..

[3] Halloran C., Ghaneh P., Bosonnet L., Hartley M., Sutton R., Neoptolemos J. (2002). Complications of pancreatic cancer resection. Digestive Surg..

[4] Sierzega M., Niekowal B., Kulig J., Popiela T. (2007). Nutritional status affects the rate of pancreatic fistula after distal pancreatectomy: A multivariate analysis of 132 patients. J. Am. Coll. Surg..

[5] Bozzetti F., Gianotti L., Braga M., Di Carlo V., Mariani L. (2007). Postoperative complications in gastrointestinal cancer patients: The joint role of the nutritional status and the nutritional support. Clin. Nutr..

[6] Kang J., Park J.S., Yoon D.S., Kim W.J., Chung H.-y., Lee S.M., Chang N. (2016). A Study on the Dietary Intake and the Nutritional Status among the Pancreatic Cancer Surgical Patients. Clin. Nutr. Res..

[7] Lassen K., Coolsen M.M., Slim K., Carli F., de Aguilar-Nascimento J.E., Schäfer M., Parks R.W., Fearon K.C., Lobo D.N., Demartines N. (2012). Guidelines for perioperative care for pancreaticoduodenectomy: Enhanced Recovery After Surgery (ERAS^®^) Society recommendations. Clin. Nutr..

[8] Gärtner S., Krüger J., Aghdassi A.A., Steveling A., Simon P., Lerch M.M., Mayerle J. (2015). Nutrition in pancreatic cancer: A review. Gastrointestinal Tumors.

[9] Keele A., Bray M., Emery P., Duncan H., Silk D. (1997). Two phase randomised controlled clinical trial of postoperative oral dietary supplements in surgical patients. Gut.

[10] Gerritsen A., Wennink R.A., Besselink M.G., Santvoort H.C., Tseng D.S., Steenhagen E., Borel Rinkes I.H., Molenaar I.Q. (2014). Early oral feeding after pancreatoduodenectomy enhances recovery without increasing morbidity. HPB.

[11] Saluja S.S., Kaur N., Shrivastava U.K. (2002). Enteral Nutrition in Surgical Patients. Surgery Today.

[12] Kang M.R. (2017). Use of Oral Nutritional Supplements for Patients with Diabetes. J. Korean Diabetes.

[13] Hubbard G.P., Elia M., Holdoway A., Stratton R.J. (2012). A systematic review of compliance to oral nutritional supplements. Clin. Nutr..

[14] Fell G.L., Nandivada P., Gura K.M., Puder M. (2015). Intravenous Lipid Emulsions in Parenteral Nutrition. Adv. Nut..

[15] Adolph M., Heller A., Koch T., Koletzko B., Kreymann K., Krohn K., Pscheidl E., Senkal M., Working group for developing the guidelines for parenteral nutrition of The German Association for Nutritional Medicine (2009). Lipid emulsions–guidelines on parenteral nutrition, chapter 6. GMS German Medical Science.

[16] Keim V., Klar E., Poll M., Schoenberg M.H. (2009). Postoperative Care Following Pancreatic Surgery: Surveillance and Treatment. Dtsch. Arztebl. Int..

[17] Schneyder A. (2014). Malnutrition and nutritional supplements. Aust. Prescriber.

[18] Blundell J.E., Macdiarmid J.I. (1997). Fat as a Risk Factor for Overconsumption: Satiation, Satiety, and Patterns of Eating. J. Am. Diet. Assoc..

[19] Nijboer B. (1957). BRA Nijboer and FW de Wette, Physica 23, 309 (1957). Physica.

[20] Trumbo P., Schlicker S., Yates A.A., Poos M. (2002). Dietary reference intakes for energy, carbohdrate, fiber, fat, fatty acids, cholesterol, protein and amino acids. J. Am. Diet. Assoc..

[21] Bouillanne O., Morineau G., Dupont C., Coulombel I., Vincent J.-P., Nicolis I., Benazeth S., Cynober L., Aussel C. (2005). Geriatric Nutritional Risk Index: A new index for evaluating at-risk elderly medical patients. Am. J. Clin. Nutr..

[22] Buzby G.P., Mullen J.L., Matthews D.C., Hobbs C.L., Rosato E.F. (1980). Prognostic nutritional index in gastrointestinal surgery. Am. J. Surg..

[23] La Torre M., Ziparo V., Nigri G., Cavallini M., Balducci G., Ramacciato G. (2013). Malnutrition and pancreatic surgery: Prevalence and outcomes. J. Surg. Oncol..

[24] Ritchie S.A., Akita H., Takemasa I., Eguchi H., Pastural E., Nagano H., Monden M., Doki Y., Mori M., Jin W. (2013). Metabolic system alterations in pancreatic cancer patient serum: Potential for early detection. BMC Cancer.

[25] Banim P.J.R., Luben R., Khaw K.-T., Hart A.R. (2018). Dietary oleic acid is inversely associated with pancreatic cancer–Data from food diaries in a cohort study. Pancreatology.

[26] Menendez J., Vellon L., Colomer R., Lupu R. (2005). Oleic acid, the main monounsaturated fatty acid of olive oil, suppresses her-2/neu (erb b-2) expression and synergistically enhances the growth inhibitory effects of trastuzumab (herceptin™) in breast cancer cells with her-2/neu oncogene amplification. Ann. Oncol..

[27] Llor X., Pons E., Roca A., Alvarez M., Mane J., Fernandez-Banares F., Gassull M. (2003). The effects of fish oil, olive oil, oleic acid and linoleic acid on colorectal neoplastic processes. Clin. Nutr..

[28] Phua L.C., Goh S., Tai D.W.M., Leow W.Q., Alkaff S.M.F., Chan C.Y., Kam J.H., Lim T.K.H., Chan E.C.Y. (2018). Metabolomic prediction of treatment outcome in pancreatic ductal adenocarcinoma patients receiving gemcitabine. Cancer Chemother. Pharmacol..

[29] Goldstein R.E. (1993). A Comparison of Medium-Chain and Long-Chain Triglycerides in Surgical Patients Z. JIANG, S. ZHANG, X. WANG, ET AL Annals of Surgery 217:175–184, 1993. J. Parenter. Enteral. Nutr..

[30] Shah N.D., Limketkai B.N. (2017). The Use of Medium-Chain Triglycerides in Gastrointestinal Disorders. Pract. Gastroenterol..

[31] Ward Dean M., English J. (2013). Medium chain triglycerides (MCTs). beneficial effects on energy, atherosclerosis and aging. Nutr. Rev..

[32] Wang X., Pan L., Zhang P., Liu X., Wu G., Wang Y., Liu Y., Li N., Li J. (2010). Enteral nutrition improves clinical outcome and shortens hospital stay after cancer surgery. J. Investing. Surg..

[33] St-Onge M.-P., Mayrsohn B., O’Keeffe M., Kissileff H.R., Choudhury A.R., Laferrère B. (2014). Impact of medium and long chain triglycerides consumption on appetite and food intake in overweight men. Eur. J. Clin. Nutr..

[34] Kawai K., Nakashima M., Kojima M., Yamashita S., Takakura S., Shimizu M., Kubo C., Sudo N. (2017). Ghrelin activation and neuropeptide Y elevation in response to medium chain triglyceride administration in anorexia nervosa patients. Clin. Nutr. ESPEN.

[35] Shen M.-C., Zhao X., Siegal G.P., Desmond R., Hardy R.W. (2014). Dietary stearic acid leads to a reduction of visceral adipose tissue in athymic nude mice. PLoS ONE.

[36] Lissner L., Levitsky D.A., Strupp B.J., Kalkwarf H.J., Roe D.A. (1987). Dietary fat and the regulation of energy intake in human subjects. Am. J. Clin. Nutr..

[37] Lawton C.L., Delargy H.J., Brockman J., Smith F.C., Blundell J.E. (2000). The degree of saturation of fatty acids influences post-ingestive satiety. Br. J. Nutr..

[38] Tordoff M.G., Reed D.R. (1991). Sham-feeding sucrose or corn oil stimulates food intake in rats. Appetite.

[39] Kühn T., Floegel A., Sookthai D., Johnson T., Rolle-Kampczyk U., Otto W., von Bergen M., Boeing H., Kaaks R. (2016). Higher plasma levels of lysophosphatidylcholine 18: 0 are related to a lower risk of common cancers in a prospective metabolomics study. BMC Med..

[40] Dong J., Cai X., Zhao L., Xue X., Zou L., Zhang X., Liang X. (2010). Lysophosphatidylcholine profiling of plasma: Discrimination of isomers and discovery of lung cancer biomarkers. Metabolomics.

[41] Goto T., Terada N., Inoue T., Kobayashi T., Nakayama K., Okada Y., Yoshikawa T., Miyazaki Y., Uegaki M., Utsunomiya N. (2015). Decreased expression of lysophosphatidylcholine (16: 0/OH) in high resolution imaging mass spectrometry independently predicts biochemical recurrence after surgical treatment for prostate cancer. Prostate.

[42] Sutphen R., Xu Y., Wilbanks G.D., Fiorica J., Grendys E.C., LaPolla J.P., Arango H., Hoffman M.S., Martino M., Wakeley K. (2004). Lysophospholipids are potential biomarkers of ovarian cancer. Cancer Epidemiol. Biomark. Prev..

[43] D’Angelo G., Moorthi S., Luberto C. (2018). Role and Function of Sphingomyelin Biosynthesis in the Development of Cancer. Adv. Cancer Res..

[44] Asano S., Kitatani K., Taniguchi M., Hashimoto M., Zama K., Mitsutake S., Igarashi Y., Takeya H., Kigawa J., Hayashi A. (2012). Regulation of cell migration by sphingomyelin synthases: Sphingomyelin in lipid rafts decreases responsiveness to signaling by the CXCL12/CXCR4 pathway. Mol. Cell. Biol..

