# Supplementary material for: Potential Nutritional and Metabolomic Advantages of High Fat Oral Supplementation in Pancreatectomized Pancreaticobiliary Cancer Patients
Source: Nutrients. 2019 Apr 20;11(4):893. doi: 10.3390/nu11040893 (PMC6521063; doi:10.3390/nu11040893)
Supplement: Supplementary file 1 [file nutrients-11-00893-s001.pdf]

Supplementary Table S1. Fatty acid composition (%) of the high fat oral supplement

	Fatty acid	% fatty acids
C18:1(n-9)	cis-Oleic acid	47.44
C8:0	Caprylic acid	19.99
C18:2(n-6)	cis-Linoleic acid	13.07
C10:0	Carpric acid	7.97
C16:0	Palmitic acid	3.77
C18:3(n-3)	α-Linolenic acid	3.51
C18:0	Stearic acid	1.85
C20:1	cis-Eicosenoic acid	0.76
C22:0	Behenic acid	0.36
C20:0	Arachidic acid	0.30
C18:2n-6	Trans-Linolelaidic acid	0.24
C24:0	Lignoceric acid	0.14
C16:1	Palmitoleic acid	0.12
C18:1(n-9)	trans-Elaidic acid	0.12
C14:0	Myristic acid	0.08
C24:1	Nervonic acid	0.07
C17:0	Heptadecanoric acid	0.06
C22:1(n-9)	Erucic acid	0.05
C20:2	cis-Eicosadienoic acid	0.05
C12:0	Lauric acid	0.04
C4:0	Butyric acid	0
C6:0	Caproric acid	0
C23:0	Tricosanoic acid	0
C22:2	cis-Docosadienoic acid	0
C22:6(n-3)	cis-Docosahexaenoic acid	0
C14:1	Myristoleic acid	0
C20:3(n-3)	cis-Eicosatrienoic acid	0
C15:0	Pentadecanoic acid	0
C15:1	cis-Pentadecanoic acid	0
C21:0	Heneicosanoic acid	0
C17:1	cis-Heptadecenoic acid	0
C18:3(n-6)	γ- Linoleic acid	0
C20:4(n-6)	Arachidonic acid	0
C20:3(n-6)	cis-Eicosatrienoic acid	0
C13:0	Tridecanoic acid	0
C20:5(n-3)	cis-Eicosapentaenoic acid	0

Supplementary Table S2. List of total metabolites differentially identified in the comparison of Non-HFS and HFS groups at preDC

m/z	RT(min)	Metabolite	HMDB	VIP	Log2FC	p-value	Superpathway	Pathway
427.2647	7.37	Chitobiose	HMDB03556	2.25	1.55	0.006	Carbohydrate	Amino sugar and nucleotide sugar metabolism
302.1209	9.56	N-Acetylglucosamine 6-sulfate	HMDB00814	1.76	1.26	0.013	Carbohydrate	Amino sugar and nucleotide sugar metabolism
176.0703	7.68	Ascorbic acid	HMDB00044	1.41	1.23	0.002	Carbohydrate	Ascorbate and aldarate metabolism
267.0582	0.8	2,3-Diphosphoglyceric acid	HMDB01294	1.07	0.33	0.044	Carbohydrate	Glycolysis / Gluconeogenesis
187.0572	0.66	2-Phosphoglyceric acid	HMDB00362	1.02	1.37	0.007	Carbohydrate	Glycolysis / Gluconeogenesis
553.2941	15.65	Lithocholate 3-O-glucuronide	HMDB02513	1.70	0.61	0.009	Carbohydrate	Pentose and glucuronate interconversions
211.1047	0.82	Vanilpyruvic acid	HMDB11714	1.49	1.36	<0.001	Carbohydrate	Pyruvate metabolism
114.0662	0.62	1-Pyrroline-5-carboxylic acid	HMDB01301	1.25	0.52	0.001	Amino acid	Alanine, aspartate and glutamate metabolism
132.1016	1.65	Hydroxyproline	HMDB00725	1.10	0.50	0.033	Amino acid	Arginine and proline metabolism
229.991	1.66	L-leucyl-L-proline	HMDB11175	1.23	0.58	0.011	Amino acid	Arginine and proline metabolism
229.1193	10.92	Prolylhydroxyproline	HMDB06695	1.44	1.27	0.011	Amino acid	Arginine and proline metabolism
241.1198	11.29	Anserine	HMDB00194	1.37	1.02	0.003	Amino acid	beta-Alanine metabolism
175.0861	1.98	Dehydroascorbic acid	HMDB01264	1.23	0.33	0.002	Amino acid	Catecholamine Biosynthesis
177.0736	7.68	N-Formyl-L-methionine	HMDB01015	1.16	1.59	0.002	Amino acid	Cysteine and methionine metabolism
130.0973	0.83	Pyroglutamic acid	HMDB00267	1.12	0.51	0.011	Amino acid	Glutathione metabolism
458.2081	7.77	5,10-Methylenetetrahydrofolic acid	HMDB01533	1.39	1.26	0.038	Amino acid	Glycine, serine and threonine metabolism
190.0766	0.67	L-beta-aspartyl-L-glycine	HMDB11165	1.32	0.89	0.018	Amino acid	Glycine, serine and threonine metabolism
224.029	5.29	L-Cystathionine	HMDB00099	1.37	0.47	0.013	Amino acid	Glycine, serine and threonine metabolism
204.0626	1.21	L-Tryptophan	HMDB00929	1.39	0.95	0.004	Amino acid	Glycine, serine and threonine metabolism
226.0679	1.07	Carnosine	HMDB00033	1.28	0.65	0.003	Amino acid	Histidine metabolism
238.9887	1.89	D-Erythro-imidazole-glycerol-phosphate	HMDB12208	1.17	0.29	0.004	Amino acid	Histidine metabolism
339.0893	12.9	Imidazoleacetic acid ribotide	HMDB06032	1.41	0.80	0.038	Amino acid	Histidine metabolism
176.1278	0.68	N-Formyl-L-glutamic acid	HMDB03470	1.10	0.70	0.015	Amino acid	Histidine metabolism
288.2169	9.71	N-Ribosylhistidine	HMDB02089	1.10	0.70	0.018	Amino acid	Histidine metabolism
160.0306	0.94	Oxoadipic acid	HMDB00225	1.31	0.71	0.015	Amino acid	Lysine biosynthesis
295.1165	13.02	Glutamylphenylalanine	HMDB00594	1.42	0.83	0.001	Amino acid	Phenylalanine metabolism
165.0542	1.21	m-Coumaric acid	HMDB01713	1.17	0.71	0.007	Amino acid	Phenylalanine metabolism
208.8096	0.58	N-Acetyl-L-phenylalanine	HMDB00512	1.26	0.24	0.033	Amino acid	Phenylalanine metabolism
262.1471	5.37	O-Phosphotyrosine	HMDB06049	1.37	1.00	0.007	Amino acid	Phenylalanine metabolism
221.19	14.44	5-Hydroxy-L-tryptophan	HMDB00472	1.29	0.63	0.044	Amino acid	Tryptophan metabolism
191.0397	0.59	5-Methoxytryptamine	HMDB04095	1.48	0.59	0.005	Amino acid	Tryptophan metabolism
301.1176	9.59	Cinnavalininate	HMDB04078	1.63	1.63	0.015	Amino acid	Tryptophan metabolism
225.0094	4.5	L-3-Hydroxykynurenine	HMDB11631	1.14	1.33	0.002	Amino acid	Tryptophan metabolism
233.1126	1.43	Melatonin	HMDB01389	1.09	0.88	0.029	Amino acid	Tryptophan metabolism
219.1745	13.8	N-Acetylserotonin	HMDB01238	1.21	0.51	<0.001	Amino acid	Tryptophan metabolism
237.0905	11.51	N'-Formylkynurenine	HMDB01200	1.19	0.72	0.001	Amino acid	Tryptophan metabolism
206.0236	5.02	Xanthurenic acid	HMDB00881	1.13	0.24	0.021	Amino acid	Tryptophan Metabolism
216.014	0.57	3-Chlorotyrosine	HMDB01885	1.13	1.01	0.006	Amino acid	Tyrosine metabolism
227.1383	4.34	3-Nitrotyrosine	HMDB01904	1.26	0.43	0.024	Amino acid	Tyrosine metabolism
282.1189	1.03	4-Hydroxyphenylacetylglutamine	HMDB06061	1.09	0.43	0.006	Amino acid	Tyrosine metabolism
183.084	1.22	Homovanillic acid	HMDB00118	1.17	0.78	0.006	Amino acid	Tyrosine metabolism
198.1234	1.17	Metanephrine	HMDB04063	1.23	0.92	0.024	Amino acid	Tyrosine metabolism
153.0518	1.88	p-Hydroxyphenylacetic acid	HMDB00020	1.17	0.62	0.002	Amino acid	Tyrosine metabolism
218.1614	13.97	Tyramine-O-sulfate	HMDB06409	1.50	1.25	0.005	Amino acid	Tyrosine metabolism
173.0917	0.65	S-(3-Methylbutanoyl)-dihydrolipoamide-E	HMDB06867	1.12	0.90	0.001	Amino acid	Valine, leucine and isoleucine biosynthesis
339.2885	16.49	14,15-Dihydroxyeicosatrienoic acid	HMDB02265	1.27	1.10	0.007	Lipid	Arachidonic acid metabolism
367.2683	11.11	20-Carboxyleukotriene B4	HMDB06059	1.00	0.27	0.013	Lipid	Arachidonic acid metabolism
319.2266	21.41	Leukotriene A4	HMDB01337	1.45	1.48	0.015	Lipid	Arachidonic acid metabolism

353.2214	8.74	Lipoxin A4	HMDB04385	2.28	2.28	<0.001	Lipid	Arachidonic acid metabolism
335.1768	11.32	Prostaglandin J2	HMDB02710	1.23	1.17	0.044	Lipid	Arachidonic acid metabolism
319.2622	16.94	14(15)-epoxy-5Z,8Z,11Z,17Z-eicosatetraenoate	HMDB10205	1.61	1.61	0.006	Lipid	Fatty acid metabolism
363.2157	10.47	19,20-Dihydroxydocosapentaenoic acid	HMDB10214	1.38	0.66	0.044	Lipid	Fatty acid metabolism
386.2911	12.7	3-Hydroxy-cis-5-tetradecenoylcarnitine	HMDB13330	1.46	1.03	0.033	Lipid	Fatty acid metabolism
371.1881	14.48	3-Oxo-4,6-choladienoic acid	HMDB00476	1.64	1.50	0.003	Lipid	Fatty acid metabolism
389.2358	11.58	5,6-Dihydroxyprostaglandin F1a	HMDB12109	1.39	1.02	0.033	Lipid	Fatty acid metabolism
314.2324	10.92	9-Decenoylcarnitine	HMDB13205	1.42	0.94	0.024	Lipid	Fatty acid metabolism
202.1801	3.59	Capryloylglycine	HMDB00832	1.77	1.33	0.038	Lipid	Fatty acid metabolism
370.2952	13.49	cis-5-Tetradecenoylcarnitine	HMDB02014	1.28	0.77	0.011	Lipid	Fatty acid metabolism
421.2187	14.34	Cyclic phosphatic acid(18:0)	HMDB07004	1.17	1.21	0.006	Lipid	Fatty acid metabolism
417.3378	13.76	Cyclic phosphatic acid(18:2)	HMDB07007	1.18	1.07	0.007	Lipid	Fatty acid metabolism
374.5782	6.89	Dodecanedioylcarnitine	HMDB13327	2.45	2.01	0.007	Lipid	Fatty acid metabolism
430.2435	7.04	Hexadecanedioic acid mono-L-carnitine ester	HMDB00712	1.67	0.92	0.011	Lipid	Fatty acid metabolism
147.0647	13.02	Isobutyrylglycine	HMDB00730	1.29	0.87	0.001	Lipid	Fatty acid metabolism
326.377	14.34	N-Oleoylethanolamine	HMDB02088	1.52	0.97	0.001	Lipid	Fatty acid metabolism
283.1222	1.04	Oleic acid	HMDB00207	1.12	0.42	0.029	Lipid	Fatty acid metabolism
299.9109	0.82	Pristanic acid	HMDB00795	1.11	0.70	0.005	Lipid	Fatty acid metabolism
419.1819	14.54	Stearidonyl carnitine	HMDB06463	1.75	1.22	0.001	Lipid	Fatty acid metabolism
427.5991	7.37	Stearoylcarnitine	HMDB00848	2.33	1.41	0.009	Lipid	Fatty acid metabolism
232.1286	1.02	Suberylglycine	HMDB00953	1.44	0.68	0.029	Lipid	Fatty acid metabolism
371.2985	13.49	Tetradecanoylcarnitine	HMDB05066	1.58	0.81	0.029	Lipid	Fatty acid metabolism
342.263	12.4	trans-2-Dodecenoylcarnitine	HMDB13326	1.13	0.71	0.021	Lipid	Fatty acid metabolism
246.2065	3.42	Valerylcarnitine	HMDB13128	2.08	1.04	0.021	Lipid	Fatty acid metabolism
561.4114	16.7	Diglyceride(14:0/18:4)	HMDB07019	1.32	0.87	0.024	Lipid	Glycerolipid metabolism
607.5663	20.54	Diglyceride(15:0/20:2)	HMDB07080	1.21	1.06	0.004	Lipid	Glycerolipid metabolism
605.5411	19.38	Diglyceride(15:0/20:3)	HMDB07081	1.27	1.07	0.002	Lipid	Glycerolipid metabolism
635.5972	21.36	Diglyceride(15:0/22:2)	HMDB07088	1.36	1.86	0.007	Lipid	Glycerolipid metabolism
569.3419	15.58	Diglyceride(16:0/16:0)	HMDB07098	1.21	0.89	0.018	Lipid	Glycerolipid metabolism
637.4938	15.65	Diglyceride(18:3/20:5)	HMDB07288	1.03	-0.48	<0.001	Lipid	Glycerolipid metabolism
663.5696	21.48	Diglyceride(18:3/22:6)	HMDB07295	2.15	3.06	0.005	Lipid	Glycerolipid metabolism
703.562	20.35	Diglyceride(18:3/24:0)	HMDB07296	1.97	2.92	0.005	Lipid	Glycerolipid metabolism
701.5581	17.97	Diglyceride(18:3/24:1)	HMDB07297	1.33	1.10	0.002	Lipid	Glycerolipid metabolism
727.5749	18.07	Diglyceride(20:4/24:1)	HMDB07529	1.36	1.39	0.006	Lipid	Glycerolipid metabolism
719.5703	18.13	Diglyceride(22:4/22:5)	HMDB07700	1.06	0.68	0.033	Lipid	Glycerolipid metabolism
437.1782	8.51	Lysophosphatidic acid(18:1)	HMDB07851	1.19	1.50	0.024	Lipid	Glycerolipid metabolism
357.299	16.49	Monoglyceride(18:1)	HMDB11536	1.32	0.87	0.011	Lipid	Glycerolipid metabolism
381.3165	13.87	Monoglyceride(20:3)	HMDB11547	1.32	1.02	0.018	Lipid	Glycerolipid metabolism
441.3321	5.15	Monoglyceride(24:1)	HMDB11559	1.39	0.34	0.044	Lipid	Glycerolipid metabolism
431.351	17.7	Monoglyceride(24:6)	HMDB11590	2.07	2.51	0.002	Lipid	Glycerolipid metabolism
800.6733	19.49	Triglyceride(14:0/16:1/18:2)	HMDB10422	1.19	0.88	0.003	Lipid	Glycerolipid metabolism
875.7099	20.78	Triglyceride(14:0/20:4/20:4)	HMDB10518	1.32	-0.66	0.021	Lipid	Glycerolipid metabolism
841.7137	20.38	Triglyceride(15:0/16:0/20:4)	HMDB11702	1.20	0.65	0.007	Lipid	Glycerolipid metabolism
927.7325	21.04	Triglyceride(18:2/18:2/22:6)	HMDB10496	1.21	-1.17	0.002	Lipid	Glycerolipid metabolism
951.741	21.36	Triglyceride(18:2/20:5/22:5)	HMDB10537	1.45	-1.05	<0.001	Lipid	Glycerolipid metabolism
495.4036	16.95	Lysophosphatidylcholine(16:0)	HMDB10382	1.53	1.12	0.007	Lipid	Glycerophospholipid metabolism
520.3402	15.62	Lysophosphatidylcholine(18:2)	HMDB10386	1.48	0.77	0.011	Lipid	Glycerophospholipid metabolism
518.3527	10.26	Lysophosphatidylcholine(18:3)	HMDB10388	1.48	1.15	0.002	Lipid	Glycerophospholipid metabolism
552.2948	15.65	Lysophosphatidylcholine(20:0)	HMDB10390	1.55	0.52	0.029	Lipid	Glycerophospholipid metabolism
550.3141	16.08	Lysophosphatidylcholine(20:1)	HMDB10391	1.50	0.98	0.015	Lipid	Glycerophospholipid metabolism
548.3453	10.83	Lysophosphatidylcholine(20:2)	HMDB10392	1.89	1.22	0.044	Lipid	Glycerophospholipid metabolism
544.3386	15.6	Lysophosphatidylcholine(20:4)	HMDB10395	1.35	1.60	0.009	Lipid	Glycerophospholipid metabolism

570.3167	8.27	Lysophosphatidylcholine(22:5)	HMDB10402	1.39	1.36	0.033	Lipid	Glycerophospholipid metabolism
568.3384	15.58	Lysophosphatidylcholine(22:6)	HMDB10404	1.15	0.83	0.021	Lipid	Glycerophospholipid metabolism
606.387	10.99	Lysophosphatidylcholine(24:1)	HMDB10406	1.08	1.41	0.038	Lipid	Glycerophospholipid metabolism
426.1948	13.08	Lysophosphatidylethanolamine(14:0)	HMDB11470	1.37	0.94	0.005	Lipid	Glycerophospholipid metabolism
454.3878	15.36	Lysophosphatidylethanolamine(16:0)	HMDB11473	1.63	0.95	0.044	Lipid	Glycerophospholipid metabolism
480.2122	7.35	Lysophosphatidylethanolamine(18:1)	HMDB11475	1.97	1.72	0.029	Lipid	Glycerophospholipid metabolism
478.3288	18.46	Lysophosphatidylethanolamine(18:2)	HMDB11477	1.06	-0.85	0.009	Lipid	Glycerophospholipid metabolism
510.451	16.13	Lysophosphatidylethanolamine(20:0)	HMDB11481	1.52	0.76	0.009	Lipid	Glycerophospholipid metabolism
508.3769	16.4	Lysophosphatidylethanolamine(20:1)	HMDB11482	1.53	0.79	0.015	Lipid	Glycerophospholipid metabolism
504.3737	16.74	Lysophosphatidylethanolamine(20:3)	HMDB11484	2.08	3.14	0.006	Lipid	Glycerophospholipid metabolism
502.2056	7.99	Lysophosphatidylethanolamine(20:4)	HMDB11487	1.84	2.30	0.004	Lipid	Glycerophospholipid metabolism
503.2826	13.14	Lysophosphatidylethanolamine(20:4)	HMDB11518	1.45	0.72	0.011	Lipid	Glycerophospholipid metabolism
538.295	10.26	Lysophosphatidylethanolamine(22:0)	HMDB11490	1.24	0.89	0.033	Lipid	Glycerophospholipid metabolism
536.3192	10.29	Lysophosphatidylethanolamine(22:1)	HMDB11491	1.29	1.16	0.029	Lipid	Glycerophospholipid metabolism
530.29	15.48	Lysophosphatidylethanolamine(22:4)	HMDB11493	1.52	1.31	0.003	Lipid	Glycerophospholipid metabolism
564.3584	8.27	Lysophosphatidylethanolamine(24:1)	HMDB11498	1.38	0.79	0.021	Lipid	Glycerophospholipid metabolism
678.5614	18.05	Phosphatidylcholine(14:0/14:0)	HMDB07866	1.40	1.28	0.004	Lipid	Glycerophospholipid metabolism
704.5651	20.35	Phosphatidylcholine(14:0/16:1)	HMDB07870	2.13	3.00	0.003	Lipid	Glycerophospholipid metabolism
779.5785	19.08	Phosphatidylcholine(14:0/22:6)	HMDB07892	1.12	-1.44	0.007	Lipid	Glycerophospholipid metabolism
800.5194	17.89	Phosphatidylcholine(18:4/20:5)	HMDB08248	1.25	-1.81	0.029	Lipid	Glycerophospholipid metabolism
856.6424	19.33	Phosphatidylcholine(20:3/22:6)	HMDB08387	1.00	-1.68	0.007	Lipid	Glycerophospholipid metabolism
894.7549	20.71	Phosphatidylcholine(20:3/24:1)	HMDB08389	1.28	-1.10	0.013	Lipid	Glycerophospholipid metabolism
746.6061	19.04	Phosphatidylethanolamine(14:0/22:1)	HMDB08842	1.07	0.82	0.004	Lipid	Glycerophospholipid metabolism
734.3328	7.99	Phosphatidylethanolamine(14:1/22:6)	HMDB08880	1.79	2.38	0.006	Lipid	Glycerophospholipid metabolism
798.6541	19.03	Phosphatidylethanolamine(18:3/22:0)	HMDB09138	1.21	0.94	0.011	Lipid	Glycerophospholipid metabolism
696.5911	21.04	Phosphatidylethanolamine(18:4/P-16:0)	HMDB09213	2.25	3.87	0.003	Lipid	Glycerophospholipid metabolism
820.6649	19.57	Phosphatidylethanolamine(20:0/22:6)	HMDB09243	1.01	-0.55	0.015	Lipid	Glycerophospholipid metabolism
814.6761	19.25	Phosphatidylethanolamine(20:3/22:6)	HMDB09342	1.14	0.71	0.003	Lipid	Glycerophospholipid metabolism
812.6154	18.98	Phosphatidylethanolamine(20:4/22:6)	HMDB09408	1.15	0.96	0.002	Lipid	Glycerophospholipid metabolism
842.7172	20.41	Phosphatidylethanolamine(22:4/22:5)	HMDB09604	1.07	0.49	0.033	Lipid	Glycerophospholipid metabolism
840.7011	19.84	Phosphatidylethanolamine(22:4/22:6)	HMDB09605	1.37	0.78	0.001	Lipid	Glycerophospholipid metabolism
876.5704	18.86	Phosphatidylethanolamine(22:5/24:1)	HMDB09674	1.37	-0.57	0.001	Lipid	Glycerophospholipid metabolism
776.6516	20.08	Phosphatidylethanolamine(22:5/P-18:1)	HMDB09677	1.18	0.23	0.029	Lipid	Glycerophospholipid metabolism
749.5317	18.53	Phosphatidylethanolamine(22:6/O-16:1)	HMDB05780	1.01	-0.83	0.004	Lipid	Glycerophospholipid metabolism
747.6013	18.03	Phosphatidylglyceride(16:0/18:2)	HMDB10575	1.57	1.42	0.001	Lipid	Glycerophospholipid metabolism
744.822	7.99	Phosphatidylglyceride(16:0/18:3)	HMDB10576	1.96	2.80	0.009	Lipid	Glycerophospholipid metabolism
802.58	16.92	Phosphatidylglyceride(18:0/20:3)	HMDB10608	1.03	-0.45	0.015	Lipid	Glycerophospholipid metabolism
867.6848	21.51	Phosphatidylinositol(16:0/20:0)	HMDB09785	1.25	-1.47	0.002	Lipid	Glycerophospholipid metabolism
676.6586	20.63	Phosphatidylserine(14:1/14:1)	HMDB12342	1.47	0.82	0.018	Lipid	Glycerophospholipid metabolism
728.5784	18.07	Phosphatidylserine(14:1/18:3)	HMDB12348	1.37	1.42	0.006	Lipid	Glycerophospholipid metabolism
780.5911	19.54	Phosphatidylserine(18:3/18:3)	HMDB12414	1.03	-3.47	0.004	Lipid	Glycerophospholipid metabolism
297.1323	13.03	13S-hydroxyoctadecadienoic acid	HMDB04667	1.40	0.90	0.003	Lipid	Linoleic acid metabolism
357.235	8.99	Tetracosahexaenoic acid	HMDB02007	1.51	0.76	0.015	Lipid	Linoleic acid metabolism
514.2303	13.95	Taurocholic acid	HMDB00036	1.37	1.17	0.033	Lipid	Primary bile acid biosynthesis
512.4666	16.4	Ceramide(d18:0/14:0)	HMDB11759	1.48	0.71	0.029	Lipid	Sphingolipid metabolism
620.4933	17.23	Ceramide(d18:1/22:1)	HMDB11775	1.41	0.73	0.029	Lipid	Sphingolipid metabolism
637.6692	21.3	Ceramide(d18:1/23:0)	HMDB00950	1.31	0.60	0.044	Lipid	Sphingolipid metabolism
946.7853	21.34	Galabiosylceramide (d18:1/22:0)	HMDB04836	1.26	-0.89	0.002	Lipid	Sphingolipid metabolism
398.3255	14.23	Phytosphingosine-1-P	HMDB12280	1.33	0.23	0.033	Lipid	Sphingolipid metabolism
731.5952	18.34	Sphingomyelin(d18:0/18:1)	HMDB12088	1.01	0.78	0.001	Lipid	Sphingolipid metabolism
801.5237	17.87	Sphingomyelin(d18:1/23:0)	HMDB12105	1.16	-1.83	0.029	Lipid	Sphingolipid metabolism
813.6184	18.98	Sphingomyelin(d18:1/24:1)	HMDB12107	1.14	0.82	0.002	Lipid	Sphingolipid metabolism

427.3572	17.47	(S)-2,3-Epoxysqualene	HMDB01188	1.63	1.04	0.018	Lipid	Steroid biosynthesis
443.6717	13.02	4a-Methylzymosterol-4-carboxylic acid	HMDB06927	1.20	0.61	0.009	Lipid	Steroid biosynthesis
489.3117	17.07	7-Sulfocholic acid	HMDB02421	1.16	0.81	0.006	Lipid	Steroid biosynthesis
383.197	15.01	Farnesyl pyrophosphate	HMDB00961	1.64	1.11	0.003	Lipid	Steroid biosynthesis
541.3289	16.06	Tetrahydroaldosterone-3-glucuronide	HMDB10357	1.44	0.70	0.029	Lipid	Steroid biosynthesis
297.1901	6.72	17a-Ethynylestradiol	HMDB01926	1.44	1.46	0.001	Lipid	Steroid hormone biosynthesis
349.2338	14.65	3b,17a,21-Trihydroxypregnenone	HMDB00382	2.10	1.41	0.001	Lipid	Steroid hormone biosynthesis
347.1592	6.79	Corticosterone	HMDB01547	1.25	1.25	0.007	Lipid	Steroid hormone biosynthesis
369.2831	12.93	Cortol	HMDB03180	1.02	0.56	0.044	Lipid	Steroid hormone biosynthesis
270.9576	0.82	Estrone	HMDB00145	1.20	1.03	0.007	Lipid	Steroid hormone biosynthesis
397.2341	15.23	Pregnenolone sulfate	HMDB00774	2.32	3.69	0.003	Lipid	Steroid hormone biosynthesis
277.0829	11.54	Biotin sulfone	HMDB04818	1.29	0.42	0.009	Cofactors and vitamins	Biotin metabolism
215.0673	7.1	Methyl bisnorbiotinyl ketone	HMDB04822	1.29	0.79	0.002	Cofactors and vitamins	Biotin metabolism
240.1433	13.97	4a-Carbinolamine tetrahydrobiopterin	HMDB02215	1.54	0.78	0.006	Cofactors and vitamins	Folate biosynthesis
256.2073	9.25	7,8-Dihydroneopterin	HMDB02275	1.33	1.56	0.033	Cofactors and vitamins	Folate biosynthesis
124.0472	1.21	Nicotinic acid	HMDB01488	1.16	0.76	0.006	Cofactors and vitamins	Nicotinate and nicotinamide metabolism
275.1459	6.15	Ribose-1-arsenate	HMDB12285	1.47	1.19	<0.001	Cofactors and vitamins	Nicotinate and nicotinamide metabolism
472.8281	16.03	5-Formiminotetrahydrofolic acid	HMDB01534	1.34	-1.01	0.001	Cofactors and vitamins	One carbon pool by folate
300.9305	0.88	9-cis-Retinoic acid	HMDB02369	1.02	-0.50	0.038	Cofactors and vitamins	Retinol metabolism
357.0849	11.83	5-Amino-6-(5'-phosphoribitylamino)uracil	HMDB03841	1.09	-0.94	0.033	Cofactors and vitamins	Riboflavin metabolism
327.1059	1.65	6,7-Dimethyl-8-(1-D-ribityl)lumazine	HMDB03826	1.36	0.48	0.018	Cofactors and vitamins	Riboflavin metabolism
266.1384	12.08	Thiamine	HMDB00235	1.91	0.79	0.002	Cofactors and vitamins	Thiamine metabolism
169.1447	3.33	Homogentisic acid	HMDB00130	1.38	0.85	0.003	Cofactors and vitamins	Ubiquinone and other terpenoid-quinone biosynthesis
182.0808	1.22	2-Methyl-3-hydroxy-5-formylpyridine-4-carboxylate	HMDB06954	1.16	0.77	0.009	Cofactors and vitamins	Vitamin B6 metabolism
220.9954	4.21	Pyridoxamine 5'-phosphate	HMDB01555	1.06	1.53	0.038	Cofactors and vitamins	Vitamin B6 metabolism
433.283	7.28	24-Hydroxycalcitriol	HMDB06228	1.08	0.63	0.038	Cofactors and vitamins	Vitamin D metabolism
294.2135	20.43	Menadione	HMDB01892	2.37	4.16	0.004	Cofactors and vitamins	Vitamin digestion and absorption
281.4364	6.9	1-Methyladenosine	HMDB03331	2.47	1.17	0.009	Nucleotide	Purine metabolism
296.1312	5.62	5-Aminoimidazole ribonucleotide	HMDB01235	1.34	1.31	0.004	Nucleotide	Purine metabolism
299.1615	12.61	7-Methylguanosine	HMDB01107	1.26	0.74	0.024	Nucleotide	Purine metabolism
136.0835	13.06	Adenine	HMDB00034	1.29	0.69	0.001	Nucleotide	Purine metabolism
268.1876	10.88	Adenosine	HMDB00050	1.17	0.69	0.038	Nucleotide	Purine metabolism
347.1482	1.64	Adenosine monophosphate	HMDB00045	1.49	0.78	0.009	Nucleotide	Purine metabolism
333.1436	9.57	2'-Deoxy-5'-inosinic acid	HMDB06555	1.85	1.76	0.002	Nucleotide	Purine metabolism
284.1855	6.89	Guanosine	HMDB00133	1.51	1.21	0.018	Nucleotide	Purine metabolism
137.0787	1.21	Hypoxanthine	HMDB00157	1.19	0.82	0.007	Nucleotide	Purine metabolism
349.0363	7.95	Inosinic acid	HMDB00175	1.06	0.73	0.003	Nucleotide	Purine metabolism
308.1761	8.54	2'-Deoxycytidine 5'-monophosphate	HMDB01202	1.24	1.36	0.033	Nucleotide	Pyrimidine metabolism
289.2202	9.69	Orotidine	HMDB00788	1.53	1.07	0.013	Nucleotide	Pyrimidine metabolism
305.3025	13.21	Thymidine 3',5'-cyclic monophosphate	HMDB01570	1.50	0.88	<0.001	Nucleotide	Pyrimidine metabolism
325.1089	1.65	Uridine 5'-phosphate	HMDB00288	1.66	0.62	0.018	Nucleotide	Pyrimidine metabolism
261.1439	5.36	L-alpha-glutamyl-L-hydroxyproline	HMDB11161	1.42	0.94	0.003	Signal transduction	HIF-1 signaling pathway
274.1834	11.99	L-Thyronine	HMDB00667	1.34	0.79	0.002	Signal transduction	Thyroid hormone signaling pathway

Metabolite identification was based on accurate mass data, retention time, experimental MS/MS spectra, and library MS/MS spectra in HMDB, MyCompoundID. Fold change (log_2-transformed) was calculated by dividing the value of metabolites in HFS preDC by Non-HFS at preDC. Calculated with Mann-Whitney *U* test for difference between values of the Non-HFS and HFS group at preDC. Pathway information was extracted from KEGG, HMDB, and SMPDB.

RT, retention time; VIP, variable importance in projection; FC, fold change

Supplementary Table S3. List of metabolites correlated with oral fat intake (kcal).

Metabolites with a positive correlation			Metabolites with a negative correlation		
Metabolites	*r*-value	*p*-value	Metabolites	*r*-value	*p*-value
Vanilpyruvic acid	0.76	0.000	Phosphatidylinositol(16:0/20:0)	-0.58	0.006
N'-Formylkynurenine	0.67	0.001	Phosphatidylethanolamine(22:6/O-16:1)	-0.57	0.007
Estrone	0.67	0.001	Phosphatidylcholine(14:0/22:6)	-0.50	0.023
Biotin sulfone	0.66	0.001	5-Formiminotetrahydrofolic acid	-0.47	0.033
17a-Ethynylestradiol	0.64	0.002	Diglyceride(18:3/20:5)	-0.46	0.034
N-Formyl-L-methionine	0.60	0.004	Lysophosphatidylethanolamine(18:2)	-0.44	0.046
Thiamine	0.60	0.004			
L-3-Hydroxykynurenine	0.59	0.005			
Methyl bisnorbiotinyl ketone	0.58	0.006			
1-Pyrroline-5-carboxylic acid	0.58	0.006			
7-Sulfocholic acid	0.56	0.008			
S-(3-Methylbutanoyl)-dihydrolipoamide-E	0.56	0.008			
Phosphatidylethanolamine(22:4/22:6)	0.54	0.011			
2-Phosphoglyceric acid	0.53	0.014			
Phosphatidylethanolamine(20:4/22:6)	0.52	0.015			
L-alpha-glutamyl-L-hydroxyproline	0.51	0.017			
Sphingomyelin(d18:1/24:1)	0.51	0.019			
6,7-Dimethyl-8-(1-D-ribityl)lumazine	0.50	0.021			
Adenosine monophosphate	0.50	0.021			
Diglyceride(15:0/20:3)	0.50	0.022			
14,15-Dihydroxyeicosatrienoic acid	0.50	0.022			
Pristanic acid	0.49	0.023			
3-Oxo-4,6-choladienoic acid	0.49	0.024			
N-Oleoylethanolamine	0.48	0.026			
3-Chlorotyrosine	0.48	0.029			
Hydroxyproline	0.48	0.029			
Prolylhydroxyproline	0.47	0.033			
Triglyceride(15:0/16:0/20:4)	0.46	0.034			
Phosphatidylglyceride(16:0/18:2)	0.46	0.036			
Phosphatidylethanolamine(14:0/22:1)	0.45	0.042			
Dehydroascorbic acid	0.45	0.043			
7-Methylguanosine	0.44	0.044			
Monoglyceride(24:6)	0.44	0.047			
Nicotinic acid	0.44	0.047			
Lysophosphatidylcholine(16:0)	0.44	0.048			
Farnesyl pyrophosphate	0.44	0.049			

Pearson's *r* correlation analysis was performed. A *p*-value of less than 0.05 was considered significant (two tailed).

Supplementary Figure S1. Heatmap of total metabolites differentially identified in the comparison of Non-HFS and HFS groups at preDC

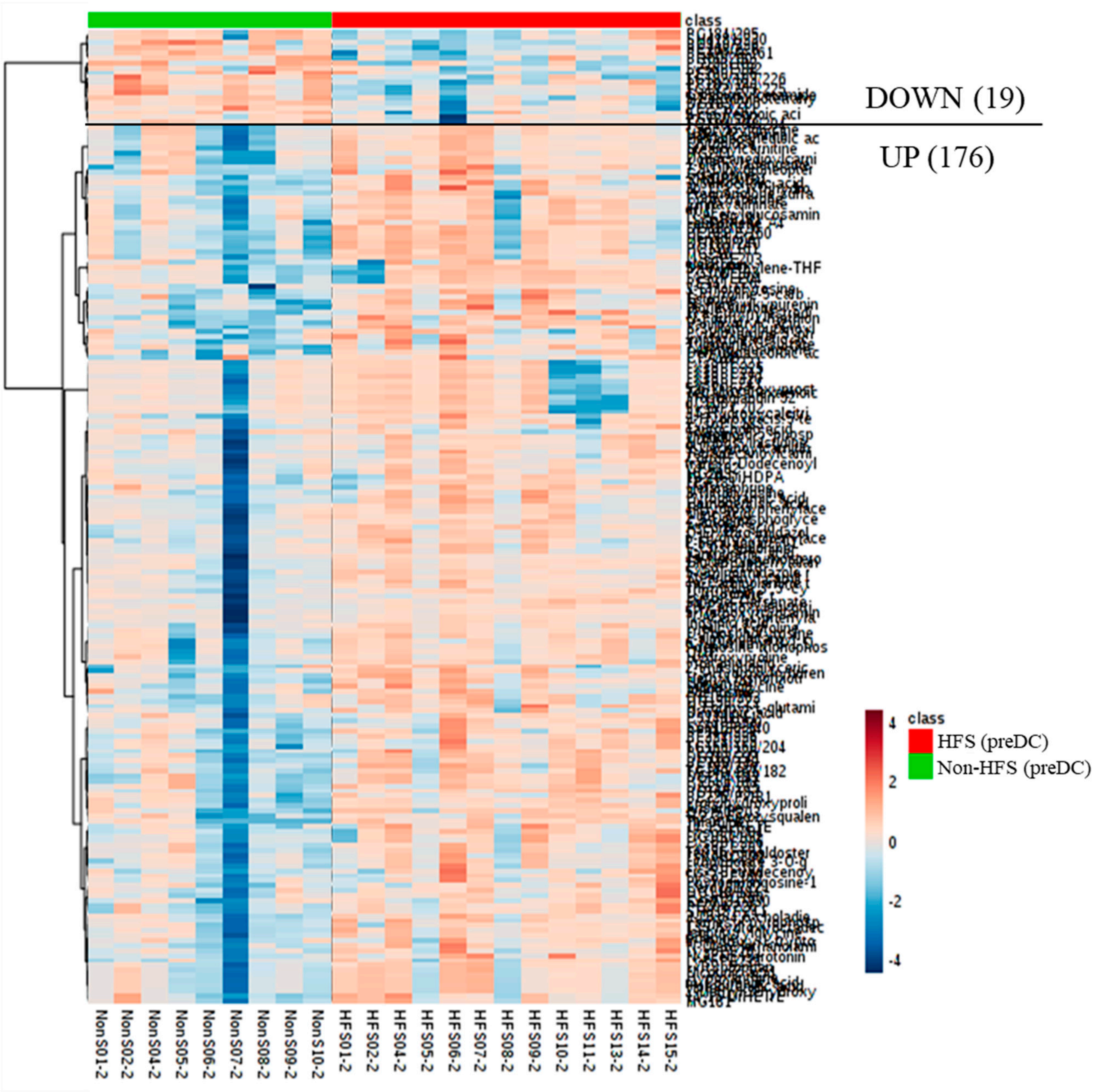

A total of 195 total metabolites were hierarchically clustered, and the metabolic changes between groups at preDC were plotted in a heat map. A total of 19 metabolites were downregulated and 176 metabolites were upregulated at preDC.

HCA, hierarchical cluster analysis; UP, upregulated; DOWN, downregulated
